# RNA Sequencing Reveals Small and Variable Contributions of Infectious Agents to Transcriptomes of Postmortem Nervous Tissues From Amyotrophic Lateral Sclerosis, Alzheimer’s Disease and Parkinson’s Disease Subjects, and Increased Expression of Genes From Disease-Activated Microglia

**DOI:** 10.3389/fnins.2019.00235

**Published:** 2019-03-28

**Authors:** James P. Bennett, Paula M. Keeney, David G. Brohawn

**Affiliations:** ^1^Neurodegeneration Therapeutics, Inc., Charlottesville, VA, United States; ^2^Parkinson’s and Movement Disorders Center, Virginia Commonwealth University, Richmond, VA, United States; ^3^Department of Medical Genetics, Virginia Commonwealth University, Richmond, VA, United States

**Keywords:** neurodegeneration, microglia, ALS, Alzheimer’s disease, Parkinson’s disease, gene expression

## Abstract

Nervous tissues from both humans with neurodegenerative diseases (NDD) and animals with genetic models of human NDD, such as rare monogenic causes of Amyotrophic Lateral Sclerosis (ALS), Alzheimer’s disease (AD), and Parkinson’s disease (PD), show activated microglia, suggesting a potential causal role for inflammation in pathogenesis of NDD. We performed paired-end (PE) RNA sequencing (RNA seq) of total RNA’s extracted from frozen sections of cervical spinal cords from ALS and CTL subjects, frontal cortical gray matter ribbons of AD and CTL subjects, and ventral midbrains of PD and CTL subjects. Trimmed PE reads were aligned against the hg38 human transcriptome using Tophat2/Bowtie2 (ALS) or HISAT2 (AD and PD) and quantitated with Cufflinks. PE reads were also aligned using Bowtie2 against genomes from representative species of *Toxoplasma gondii* and *Trichinella* sp. T6 (parasitic infectious agents), *Babesia microti* and *Borrelia burgdorferi* (tick-vector borne agents), and *Treponema denticola* and *Porphyromonas gingivalis*, agents causing chronic gingivitis. Primary aligned reads of each agent in each tissue sample were quantitated with SAMtools. We found small percentages (<0.1%) of transcriptomes aligned with *B. microti*, *B. burgdorferi*, *T. denticola*, and *P. gingivalis* genomes and larger percentages aligned with *T. gondii* (0.1–0.2%) and *Trichinella* sp. T6 (1.0–1.1%) genomes. In AD specimens, but in no others, primary aligned transcriptome percentages, although small, approached significance for being greater in AD compared to CTL samples for *B. burgdorferi* (*p* = 0.067) and *P. gingivalis* (*p* = 0.068). Genes’ expressions in postmortem tissues of AD and ALS but not PD revealed significant changes among disease-associated microglial (DAM) genes. Infectious agents’ transcripts can be detected in RNA seq reads of both NDD and CTL tissues and vary from agent to agent. Expressions of Stage 1 and Stage 2 DAM genes significantly changed, suggesting the presence of Stages 1 and 2 DAM in our NDD tissue samples.

## Introduction

Microglia are CNS-resident immune cells that can serve both beneficial (reduction of immune responses) and detrimental (activation of neurotoxic immune responses) functions (all references cited are restricted to those of last 3 years, 2016–2018) (Asiimwe et al., 2016; Beamer et al., 2016; Calsolaro and Edison, 2016; Casas et al., 2016; Chen S.H. et al., 2016; Chen W.W et al., 2016; Puentes et al., 2016; Ransohoff, 2016; Rothaug et al., 2016; Su et al., 2016; Toledano et al., 2016; Trias et al., 2016; Ulrich and Holtzman, 2016; Wes et al., 2016; Au and Ma, 2017; Bagyinszky et al., 2017; Bickford et al., 2017; Blank and Prinz, 2017; Blaylock, 2017; Bolos et al., 2017; Cerami et al., 2017; Clayton et al., 2017; Collier et al., 2017; Colonna and Butovsky, 2017; Du et al., 2017; Guerriero et al., 2017; Han et al., 2017; Herz et al., 2017; Jay et al., 2017; Joers et al., 2017; Keren-Shaul et al., 2017; Kober and Brett, 2017; Koellhoffer et al., 2017; Labandeira-Garcia et al., 2017; Lall and Baloh, 2017; Lannes et al., 2017; Nissen, 2017; Plaza-Zabala et al., 2017; Roser et al., 2017; Sochocka et al., 2017; Sorce et al., 2017; Spittau, 2017; Thompson and Tsirka, 2017; Tse, 2017; van Horssen et al., 2017; Wolf et al., 2017; Yan et al., 2017; Yang et al., 2017; Aguilera et al., 2018; Baufeld et al., 2018; Bisht et al., 2018; Crisafulli et al., 2018; Deczkowska et al., 2018; Edison and Brooks, 2018; Labzin et al., 2018; Maccioni et al., 2018; Niranjan, 2018; Selles et al., 2018; Solleiro-Villavicencio and Rivas-Arancibia, 2018; Spagnuolo et al., 2018; Taylor et al., 2018).

Because activated microglia can produce known neurotoxic substances, such as tumor necrosis factor alpha (TNF-α) (Asiimwe et al., 2016; Islam, 2017; Tse, 2017), microglial presence has suggested that immune-mediated neurodegeneration may contribute to disease origin and/or progression in human neurodegenerative diseases (NDD) (*op* cit above).

By sorting brain immune cells and carrying out massively parallel RNA sequencing (RNA seq) on these cells over the course of disease progression in the 5X FAD mouse model of human AD Keren-Shaul et al. (2017), demonstrated the TREM2-independent (“Stage 1”) and subsequent TREM2-dependent (“Stage 2”) emergence of “disease-associated microglia,” or DAM, during clinical and pathological progression (TREM = “triggering receptor expressed on myeloid cells”).

Such DAM appeared to originate from “homeostatic” microglia (see Figure 6 in Keren-Shaul et al., 2017), then due to unknown causes, progressed to Stage 1 DAM by TREM2-independent mechanisms, followed by TREM2-mediated progression into Stage 2 DAM. At Stages 1 and 2, DAM exhibited unique genotypes, consisting mainly of up-regulated genes. Deczkowska et al. (2018) subsequently reviewed the field of DAM.

We acquired postmortem samples of CNS tissues from sporadic NDD cases and carried out moderate-high density PE RNA sequencing on total RNA to seek systems biology understandings of disease pathogenesis in ALS, AD, and PD (Bennett et al., 2016; Brohawn et al., 2016; Bennett and Keeney, 2017; Ladd et al., 2017) (see also^1^).

We now sought to query these data to test the hypothesis that subclinical CNS infections with common agents could be associated with microglial activation and presence of DAM. To do so, we sought (using the Bowtie2 aligner) to determine if any of the PE RNA seq reads aligned with bacteria or parasite genomes downloaded from the NIH genome site. We then assayed the expression in each NDD tissue sample (of CTL) of genes associated with homeostatic microglia, Stage 1 or Stage 2 DAM as defined by Keren-Shaul et al. (2017).

## Results

**Figure 1:**
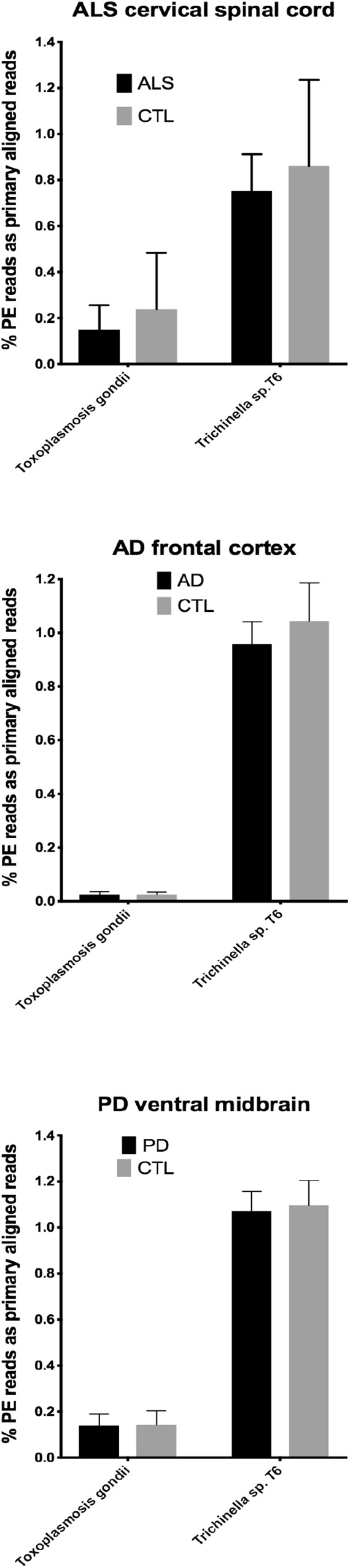
Bar charts of % of total PE reads that aligned in Bowtie2 to the genomes of *Babesia microti*, *Borrelia burgdorferi*, *Porphyromonas gingivalis*, and *Treponema denticola*. Shown are mean % +SD.

**Figure 2:**
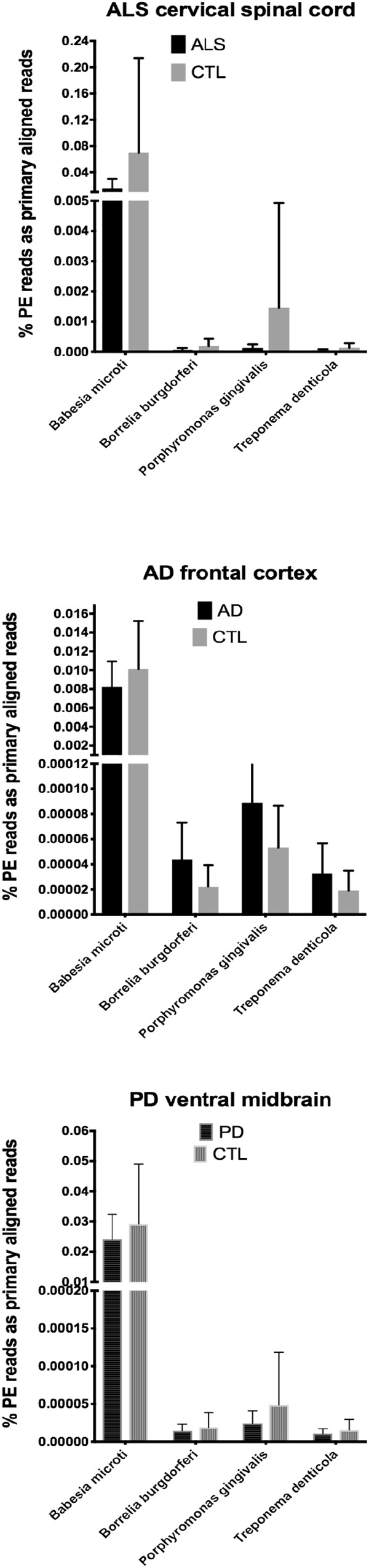
Bar charts of % of total PE reads that aligned in Bowtie2 to the genomes of *Toxoplasmosis gondii* and *Trichinella* sp. T6. Shown are mean % +SD.

In our RNA seq studies we obtained between ∼56 and ∼172 million PE reads (based on Bowtie2 alignments). From these PE reads, we found wide variation in the number of reads primarily aligned with infectious agents’ genomes. When expressed as % of total PE reads, we observed between ∼3.2 × 10^-6^ (*B. microti*) and ∼1.1 (*Trichinella* sp. T6). These results are summarized in Figure 1, 2, which show mean % aligned reads in each of the three NDD tissue specimens, expressed as mean ±SEM. In the case of AD frontal cortex samples, but in no others, we observed a difference between AD and CTL cases for *Borrelia burgdorferi* and *Porphyromonas gingivalis* that approached significance (*p* = 0.067 for *B. burgdorferi* and *p* = 0.068 for *P. gingivalis*, both by unpaired *t*-test). In no other pair did we observe a situation where NDD >CTL for transcript abundance of infectious disease agents. Because all of our total RNA extracts were treated with DNAase and used cDNA’s generated for multiplex RNA seq reads, we are confident that alignments represent NDD tissue transcripts (i.e., RNA) aligned to infectious agent genomes (i.e., DNA). We did note a substantial difference in abundance of transcripts aligned to the genome of *Trichinella* sp. T6 compared to all others examined.

**Figure 3:**
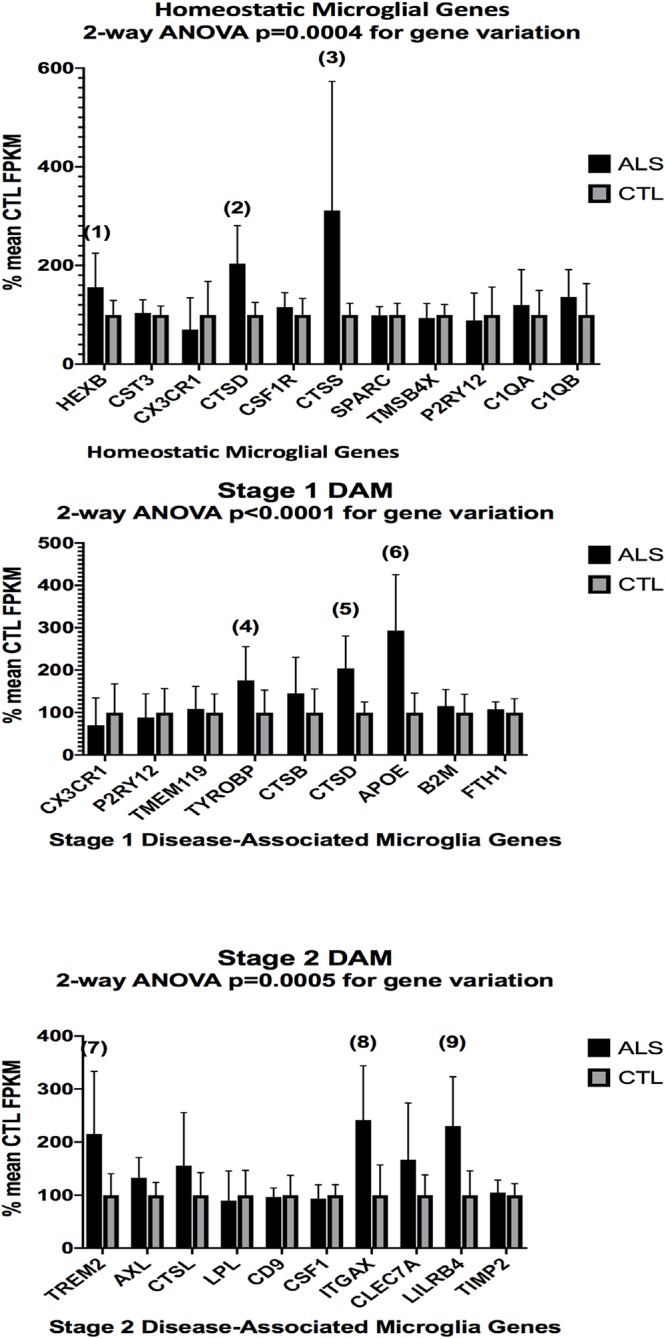
Expression of genes associated with homeostatic microglia (top graph), Stage 1 DAM (middle graph), and Stage 2 DAM (bottom graph) according to Keren-Shaul et al. (2017) in ALS and CTL cervical spinal cord samples. Gene alignments were based on paired-end (PE) Illumina sequencing of rRNA-depleted tissue total RNA, followed by removal of sequencing adapters (Trimmomatic^®^) and alignment against the hg38 version of the human genome using Tophat2/Bowtie2 and quantitation with Cufflinks. All samples with mean CTL FPKM <2.0 were removed. Data are expressed as average ±SD of mean CTL gene expression. Two-way ANOVA results are shown in each graph. Unpaired *t*-tests for significance showed: (1), *p* = 0.055 (nearly significant); (2), *p* = 0.0032; (3) *p* = 0.040; (4), *p* = 0.046; (5), *p* = 0.0032; (6), *p* = 0.0018; (7), *p* = 0.021; (8), *p* = 0.0050; (9), *p* = 0.0038. *n* = 7 AlS and 8 CTL.

**Figure 4:**
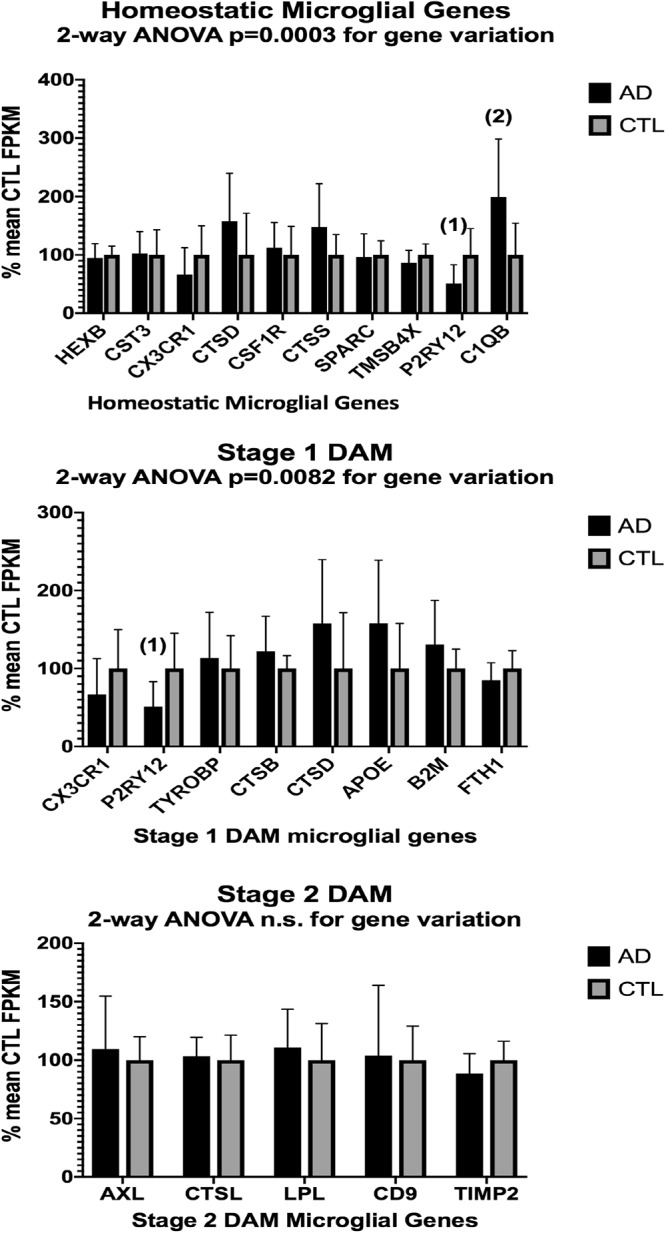
Same as Figure 3, except for AD (*n* = 10) and CTL (*n* = 9) frontal cortical ribbons. Alignments against the hg38 human genome were carried out with HISAT2 and quantitated with Cufflinks. Two-way ANOVA results are given in each graph. Unpaired *t*-test results are as follows: (1), *p* = 0.014; (2), *p* = 0.016.

**Figure 5:**
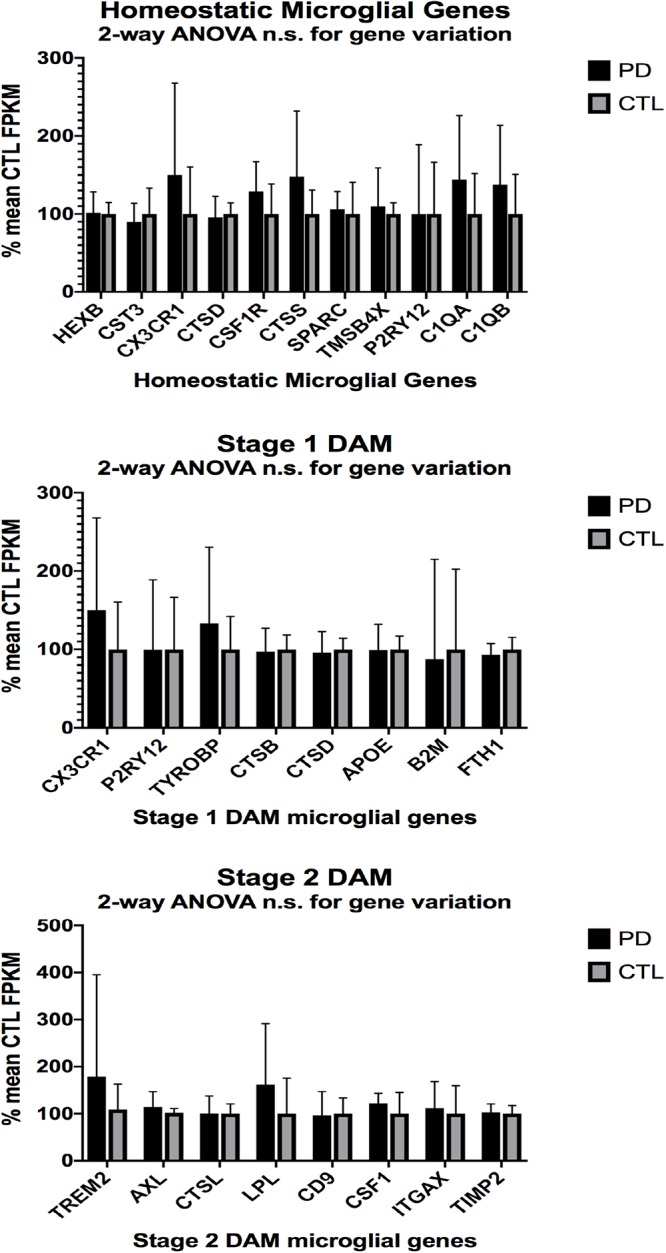
Same as Figure 3, 4, except for PD (*n* = 12) and CTL (*n* = 8) ventral midbrain. No significant differences were detected by 2-way ANOVA or unpaired *t*-tests.

In the second part of our study, we examined expressions of genes associated with homeostatic microglia and Stage 1 and Stage 2 disease-associated microglia (DAM), as defined by Keren-Shaul et al. (2017); see their Figure 6) and discussed by Deczkowska et al. (2018). Figure 3 (ALS), Figure 4 (AD), and Figure 5 (PD) show the results of our RNA seq studies of gene expressions associated with (a) homeostatic microglial genes (top graph in each Figure); (b) expression of Stage 1 DAM genes (middle graph in each Figure); and (c) expression of Stage 2 DAM genes (lower graph of each Figure). Each gene’s expression is shown as % of mean CTL tissue samples expression (±SEM) for each postmortem tissue. ALS gene expressions were derived from a Tophat2/Bowtie2/Cufflinks pipeline and the AD and PD expressions were derived from a HISAT2/Cufflinks pipeline. In all cases the genes were aligned against the most current available (hg38) version of the human genome.

Two-way ANOVA tests showed significant variation by gene for the ALS (homeostatic microglial, Stages 1 and 2 DAM) and AD (homeostatic microglial and Stage 1 DAM) samples but not for the PD samples. Individual unpaired t-tests revealed significant changes for all gene groups in the ALS and two gene groups in the AD, but none for the PD samples. We note that for the Stage 1 DAM analysis in the AD samples, we found a significant decrease in expression of P2RY12 (purinergic receptor P2Y12), also observed by Keren-Shaul et al. (2017).

## Discussion

By querying ∼52 to ∼172 million RNA seq paired-end (PE) reads in tissues from the three major adult NDD’s, we found that highly variable numbers of primary alignments could be found for several infectious agents known to affect humans. This is a limited list of infectious agents, and we appreciate that others could have been selected. These agents were selected based on documented infections in humans and availability of genomes. CNS involvement with parasitic infections, in particular, is known for *Toxoplasma* and *Trichinella* (Dzikowiec et al., 2017).

Our initial study of this approach yielded both very low frequencies of alignments (for *B. burgdorferi*, *P. gingivalis*, and *Treponema denticola*) and much higher alignment frequencies (for *Trichinella* sp. T6). With one exception (AD), we found no evidence that alignment frequencies in a NDD tissue set was greater than in CTL tissues from the same brain region. These findings suggest it is unlikely that sub-clinical infections could account for inflammation associated with NDD tissues we examined with RNA-seq. This assessment is tempered by the possibility that RNA species from the infectious agents could have been produced earlier in the NDD illnesses we studied, since we were restricted to CNS tissues from end-stage diseases.

By comparing expression of genes reported by Keren-Shaul et al. (2017) to be associated with homeostatic microglia, or Stage 1 or Stage 2 DAM, we observed significant changes in our AD and ALS but not PD populations. These findings suggest but do not prove that homeostatic microglia and DAM are present in our postmortem tissue samples. More stringent proof of homeostatic microglia or DAM existence would require RNA seq of individually identified microglia in each tissue sample. This approach is not feasible with our current logistical and economic constraints.

There are multiple limitations to our study. These include:

1. *Use of postmortem materials*. RNA seq is always problematic in these tissues, likely due to variable post-mortem intervals, inevitable RNA decay during frozen sectioning, and other unknown variations. We did attempt to use comparable RNA quality specimens, but in our hands these are always less than optimal (compared, for instance, to freshly isolated cells in culture).
2. *End-stage disease*. We do not know the effects of end-stage disease, compared to earlier stages, on any of the variables we examined. For instance, we do not know about potential loss of infectious agents’ transcripts as disease progresses, nor do we know anything about expression of DAM genes over the course of illness in humans (compared to that in mice expressing mutated NDD genes).
3. *Dilution of microglial gene expressions*. Microglia, if present, likely represent a minority of cells. As such, their contributions to total gene expression are predicted to be limited.
4. *Causal relationship(s) of Stage 1/Stage 2 DAM to neurodegeneration in each NDD*. We do not presume to ascribe causality of Stage 1/Stage 2 DAM presence to the neurodegenerative process represented by the subjects who donated tissues we used. This is particularly of concern since we were not able to define clearly any potential causes for NDD phenotype or DAM gene expression.

In spite of the above limitations, we hope that our findings will stimulate additional investigations into the potential role of DAM in pathogenesis of NDD’s. Lessening of DAM appearance or transition from Stage 1 DAM to Stage 2 DAM (Keren-Shaul et al., 2017) may represent a therapeutic opportunity in NDD. In addition, if our results can be extrapolated to multiple NDD’s, they suggest a common qualitative mechanism that could be therapeutically approached.

## Materials and Methods

Our methods for tissue acquisition, RNA seq analyses and bioinformatics have been described in multiple publications (Bennett et al., 2016; Brohawn et al., 2016, 2018; Bennett and Keeney, 2017; Ladd et al., 2017). The particular tissue sets for ALS (Bennett et al., 2016; Brohawn et al., 2016; Ladd et al., 2017), AD (Bennett and Keeney, 2017)^2^ and PD (see text footnote 1) have been previously described. Briefly, tissues from persons with sporadic NDD’s were stored at -80 degrees and blocks dissected from these unfixed, frozen specimens. Frozen 20-micron tissue sections from these unfixed tissue blocks were placed into Qiazol buffer and stored at -80 degrees until RNA isolation was carried out (miRNeasy, Qiagen). An on-column DNAase step was included for each sample. RNA quality was analyzed by gel electrophoresis. Illumina sequencing libraries were constructed according to manufacturer instructions and quantitated by qPCR, either by us (ALS/CTL cervical spinal cord) or by Cofactor Genomics (AD/CTL frontal cortex, PD/CTL ventral midbrain) and paired-end (PE) Illumina sequencing was carried out by Cofactor Genomics, Inc.^3^ Compressed (gz) PE reads in fastq format were trimmed of Illumina sequence adapters (Trimmomatic^®^) and analyzed for expression based on the hg38 human genome by Tophat2/Bowtie2 (ALS) or HISAT2 (Kim et al., 2015) and quantitated with Cufflinks. In other experiments, trimmed PE reads were aligned using Bowtie2 against genomes of infectious agents, downloaded in FASTA format from the NIH website^4^ “genome.” Bowtie2-build was used to construct Bowtie2 index files for each genome, samtools was used to convert the SAM files to BAM files, and the samtools command **samtools** **view -c -F 260 x.bam** was used to quantitate the number of primary aligned reads in each sample for each NDD. All bioinformatics assays were performed “blind” and were based solely on sample number ID (not disease state). All graph constructions, correlations and statistical assays were performed in Prism 7^5^.

## Data Availability

All tissues were acquired commercially from National Disease Resource Interchange (http://ndriresource.org; NDRI) (ALS/CTL cervical spinal cords); under the auspices of an IRB-approved collection protocol (most AD/CTL and some PD/CTL), or were declared exempt from IRB oversight (some AD/CTL and some PD/CTL). All sequencing data discussed are the property of Neurodegeneration Therapeutics, Inc., and were acquired with private funds. Untrimmed, compressed (gz) FASTQ sequencing files are available to all legitimate investigators, following request to the corresponding author (JB), completion of a Material Transfer Agreement and provision of either a FTP URL or a memory storage device capable of storing 2 TB of data. Trimmed, processed BAM files following Trimmomatic and Tophat2/Bowtie2 analyses are also available upon reasonable request.

## Author Contributions

JB, PK, and DB designed all the studies. PK oversaw the tissue acquisition and storage, and isolated and assayed the RNA’s. DB isolated the RNA’s (ALS samples), created and assayed the sequencing libraries (ALS), and performed the data analysis. JB performed all the bioinformatics assays and data analysis, and drafted the manuscript. All authors have reviewed the final manuscript and agreed with its contents.

## Conflict of Interest Statement

The authors declare that the research was conducted in the absence of any commercial or financial relationships that could be construed as a potential conflict of interest.

## Footnotes

1 http://ndtherapeutics.org/rna-sequencing-of-parkinsons-ventral-midbrain-to-estimate-gene-expression-reveals-marked-heterogeneity-and-gadd45a-as-a-potential-therapeutic-target/

2 http://ndtherapeutics.org/rna-sequencing-reveals-similarities-and-differences-in-gene-expression-in-vulnerable-brain-tissues-of-alzheimers-and-parkinsons-diseases/

3 https://cofactorgenomics.com/

4 https://www.ncbi.nlm.nih.gov/pubmed/

5 http://www.graphpad.com
